# A case of primary infertility and a patient with ventriculoperitoneal shunt

**DOI:** 10.1093/jscr/rjac590

**Published:** 2022-12-17

**Authors:** Elior Eliasi, Adiel Cohen, Oren Wasser, Gabriel Oelsner

**Affiliations:** Department of Obstetrics and Gynecology, Mayanei Hayeshua Medical Center, Sackler School of Medicine, Tel Aviv University, Bnei Brak, Israel; Department of Obstetrics and Gynecology, Hadassah Medical Organization and Faculty of Medicine, Hebrew University, Jerusalem, Israel; Hadassah-Hebrew University Medical School, Jerusalem, Israel; Department of Obstetrics and Gynecology, Mayanei Hayeshua Medical Center, Sackler School of Medicine, Tel Aviv University, Bnei Brak, Israel

## Abstract

Ventriculoperitoneal (VP) shunts are the main treatment modality for patients with hydrocephalus. The complications arising in patients with VP shunts are well documented. We present a case of infertility in a patient with a long-standing VP shunt. Her infertility is thought to be the result of pelvic adhesions due to factors related to the distal end of her VP shunt. A 22-year-old female with a 1-year history of infertility was referred due to bilateral hydrosalpinges. Diagnostic laparoscopy revealed multiple adhesions surrounding the distal end of the fallopian tubes. The distal portion of a VP shunt, which was placed over the course of her childhood, was found to be grossly inflamed and densely adherent to the pelvic viscera. VP shunts may produce abdominal adhesions and can cause mechanical infertility.

## INTRODUCTION

Ventriculoperitoneal (VP) shunting of cerebrospinal fluid (CSF) is the standard therapy for the management of hydrocephalus. Among the complications of this procedure are omental adhesions, infection, pseudocyst formation, intestinal obstruction, visceral perforation and shunt migration [1]. The incidence of complications from VP shunts has been reported in retrospective analysis at 26.7% [2]. Abdominal and genitourinary complications may occur after the shunting procedure usually due to abnormal accumulation of CSF or perforation by the shunt catheter tip [3]. An increasing number of women with CSF shunts are surviving to child-bearing age and desire to conceive. There are presently several reports regarding the occurrence and outcome of pregnancy in patients with VP shunts [4]. Two case reports have previously been published implicating VP shunts as a cause for infertility [5]. Our report describes an additional case of a patient suffering from infertility associated with salpingitis which may have been related to her VP shunt.

## CASE REPORT

As part of the investigation of a 1-year history of primary infertility, a 22-year-old patient was evaluated by trans-vaginal ultrasound. The scan showed bilateral cystic structures with the dimensions 5 by 8 cm, which were findings suitable for a diagnosis of bilateral hydrosalpinges. The uterus and ovaries appeared to be normal, and no fluid was seen in the pouch of Douglas. Previous Pap smears had been normal and there was no history of pelvic inflammatory disease or sexually transmitted diseases. The patient was born at 32 weeks after placental abruption. A month after her birth she was diagnosed with hydrocephalus, and a VP shunt was placed. The patient had not undergone any further surgeries, including abdominal surgery of VP shunt revision. The patient received no medications, had no allergies and denied tobacco, alcohol or drug abuse. Menarche occurred at 12 years of age.

Physical examination revealed a normal weight healthy female (body mass index = 23) with a blood pressure of 108/64. There was no thyromegaly or lymphadenopathy. The abdomen was soft, non-tender, not distended and without hepatosplenomegaly. On pelvic exam, the uterus was at mid-position, normal in size and not tender. No masses were palpated in the adnexa.

**Figure 1:**
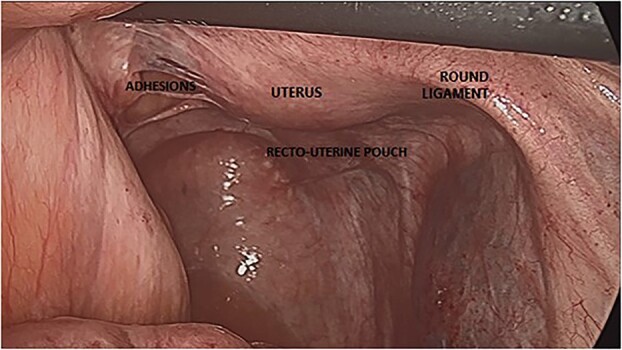
Posterior view of the uterus during laparoscopy.

As part of infertility evaluation, Diagnostic laparoscopy showed dense adhesions throughout the pelvic viscera, involving the uterus, fallopian tubes and ovaries (Fig. 1). The anterior portion of the uterus was adherent to the abdominal wall by dense adhesions. The distal portions of both fallopian tubes were embedded in dense adhesions. Both ovaries could not be seen, as they were embedded in dense adhesions. The VP shunt appeared to be intact and showed no sign of infection or other abnormalities. There was no sign of acute infection in the abdominal or pelvis. Some adehesiolysis of the pelvic region was performed. Considering the severe and organized pelvic adhesions, there was no place to perform neosalpingostomy. The laparoscopy was without any complication. The post-operative period was uneventful, and the patient was referred to *in vitro* fertilization (IVF) treatment. After a number of courses of IVF, the patient successfully became pregnant.

## DISCUSSION

Extensive pelvic adhesions may cause mechanical infertility. Our patient had no other apparent factor that could explain her infertility except for abdominal and pelvic adhesions.

In patients with a VP shunt, possible mechanisms for peritoneal adhesions include foreign body reaction and peritonitis. Although considered as a rare complication of VP shunts, spontaneous bacterial peritonitis from sterile CSF can occur [6]. Peritoneal irritation by CSF has been reported to lead to the formation of peritoneal pseudocysts without peritonitis [7]. The technique used to insert the catheter and the site of insertion could play a role in the formation of pelvic adhesions, although no direct evidence has been reported. Several reports exist describing catheter can cause perforation of the bowel and rectum and protrusion through the umbilicus, suggesting that local complications may be caused by the catheter itself [8].

## CONCLUSIONS

In our patient, diagnostic laparoscopy was performed as part of her infertility investigation in order to establish her mechanical infertility. However, in women with VP shunts, laparoscopy can be dangerous due to the severe pelvic adhesion and the risk for bowel injury. Open laparoscopy technique should be applied in these cases. The treatment of choice in patients with severe adhesion should be IVF.

## CONFLICT OF INTEREST STATEMENT

The authors certify that there is no conflict of interest with any financial organization regarding the material discussed in the manuscript.

## FUNDING

None.
